# Liquid biopsy prediction of axillary lymph node metastasis, cancer recurrence, and patient survival in breast cancer: A meta-analysis

**DOI:** 10.1097/MD.0000000000012862

**Published:** 2018-10-19

**Authors:** Ju-Han Lee, Hoiseon Jeong, Jung-Woo Choi, Hwa Eun Oh, Young-Sik Kim

**Affiliations:** Department of Pathology, Korea University Ansan Hospital, Ansan-Si, Gyeonggi-Do, Republic of Korea.

**Keywords:** breast cancer, liquid biopsy, lymph node metastasis, meta-analysis, survival

## Abstract

**Background:**

Liquid biopsies using circulating tumor DNA (ctDNA) and cell-free DNA (cfDNA) have been developed for early cancer detection and patient monitoring. To investigate the clinical usefulness of ctDNA aberrations and cfDNA levels in patients with breast cancer (BC), we conducted a meta-analysis of 69 published studies on 5736 patients with BC.

**Methods:**

The relevant publications were identified by searching PubMed and Embase databases. The effect sizes of outcome parameters were pooled using a random-effects model.

**Results:**

The ctDNA mutation rates of *TP53, PIK3CA,* and *ESR1* were approximately 38%, 27%, and 32%, respectively. High levels of cfDNA were associated with BCs rather than with healthy controls. However, these detection rates were not satisfactory for BC screening. Although the precise mechanisms have been unknown, high cfDNA levels were significantly associated with axillary lymph node metastasis (odds ratio [OR] = 2.148, *P* = .030). The ctDNA mutations were significantly associated with cancer recurrence (OR = 3.793, *P* < .001), short disease-free survival (univariate hazard ratio [HR] = 5.180, *P* = .026; multivariate HR = 3.605, *P* = .001), and progression-free survival (HR = 1.311, *P* = .013) rates, and poor overall survival outcomes (HR = 2.425, *P* = .007).

**Conclusion:**

This meta-analysis demonstrates that ctDNA mutation status predicts disease recurrence and unfavorable survival outcomes, while cfDNA levels can be predictive of axillary lymph node metastasis in patients with BC.

## Abstract 2

Supplemental Digital Content is available in the text

## 1 Introduction

Liquid biopsies using circulating tumor DNA (ctDNA) and cell-free DNA (cfDNA) have shown great potential as biomarkers for early detection, drug resistance, tumor relapse, and for predicting clinical outcomes in patients with cancer.^[1,2]^ The ctDNA originates mainly from apoptotic and necrotic cancer cells, which release fragmented DNA into the circulation.^[1,2]^ The ctDNA is known to be a specific type of cell-free DNA (cfDNA), which may also be released by dying nonmalignant host cells. The alterations in ctDNA include aberrant mutations, hypermethylation, and copy number variations.^[1,2]^ With advances in molecular diagnostics, clinicians can screen ctDNA and cfDNA for monitoring patients with cancer. Liquid biopsies monitoring ctDNA or cfDNA are expected to be superior to currently widely used plasma biomarkers, such as cancer-implanted antigen, cancer antigen 15-3 (CA15-3), and cancer antigen 19-9 (CA19-9), in terms of test's sensitivity and clinical correlations.

Breast cancer (BC) is the most common cancer in women worldwide. There have been many efforts to find better biomarkers for early detection and treatment monitoring.^[2]^ Liquid biopsy studies using ctDNA and cfDNA have been conducted in patients with BC.^[3–71]^ However, the clinical sensitivity and specificity of ctDNA or cfDNA is unsatisfactory probably due to the complex genetic heterogeneity of BC. The issue of whether a panel of genes should be tested for liquid biopsy has become very important. The prediction of axillary lymph node metastasis has an important factor on whether postoperative adjuvant chemotherapy should be performed. However, no consensus has been reached regarding the prediction of axillary lymph node metastasis or cancer recurrence using liquid biopsy in patients with BC. Therefore, the clinical utility of liquid biopsy using ctDNA and cfDNA in patients with BC has not yet been established.

To address these problems, we investigated the clinical utility of liquid biopsy to analyze ctDNA mutations or hypermethylation and cfDNA levels in patients with BC by conducting a meta-analysis of existing primary studies.

## 2 Methods

### 2.1 Selection of published studies

**Figure 1:**
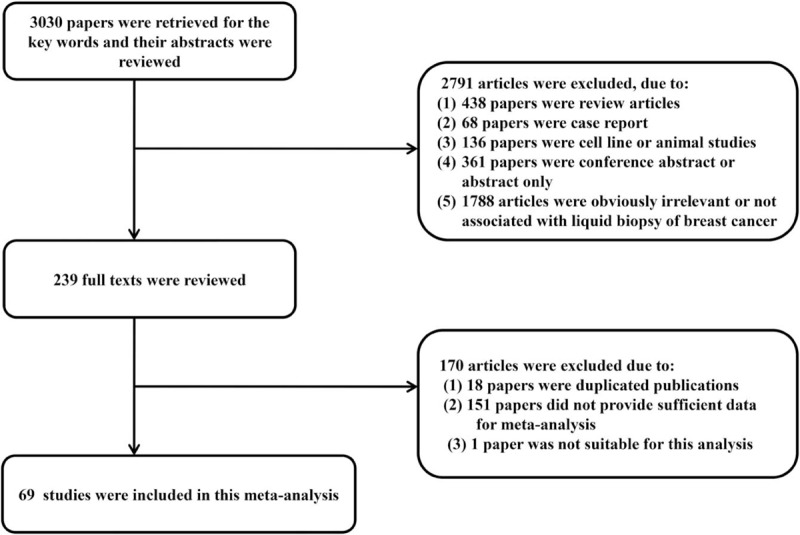
Flow diagram for article selection.

Systematic literature searches of PubMed (http://www.ncbi.nlm.nih.gov/pubmed) and Embase (www.embase.com) databases were conducted. The search strings were generated by combining keywords “circulating cell-free DNA” or “plasma cell-free DNA” or “serum cell-free DNA” or “liquid biopsy,” and “breast cancer” or “breast neoplasm.” The selection process of the articles is shown in Figure 1. All the eligible studies were reviewed and extracted independently by 2 authors (JHL and YSK), and the disagreement was resolved by discussion. There were no geographic or language restrictions. We also manually searched through the reference lists of the identified articles. The ethical approval and patient consent are not required, because this study is a meta-analysis of previously published studies.

### 2.2 Selection criteria

Original articles published before February 2017 were included. We retrieved 3030 articles using the keywords. Articles review articles without original data, conference abstracts, case reports, and articles that dealt with cell line or animal studies were excluded. We also excluded the articles that are not explicitly related to the subject. After the excluding process, the full text of 239 articles was thoroughly reviewed. Duplicate data or overlapping articles were excluded by examining author names and affiliations. When multiple articles were published by the same authors or institutions, the most recent or single informative article was selected. We also excluded the articles that provided insufficient data for meta-analysis. As a result, 69 studies were included in this meta-analysis.

### 2.3 Data extraction and quality assessment

The following data were extracted from each study: sample size, ethnicity, detection methods, liquid biopsy results, and clinicopathologic parameters including axillary lymph node metastasis, clinical stage, tumor recurrence, and survival data. The quality of the selected studies was assessed with the Newcastle–Ottawa scales (NOSs).

### 2.4 Statistical analysis

Meta-analysis was performed as previously described.^[72]^ The effect sizes and 95% confidence intervals (CIs) of each study were calculated using the inverse variance method and combined using the random-effects model (DerSimonian–Laird method). The choice of model was based on a conceptual understanding of whether the studies included in the meta-analysis, rather than homogeneity tests, all share the same population effect size.^[73]^ The summary effects were presented as the prevalence rate, odds ratio (OR; stage, axillary lymph node metastasis, and recurrence), weighted mean difference (WMD; cfDNA levels between controls and patients with BC), or HR (survival data). Heterogeneity among studies was evaluated using the Cochrane *Q* test and *I*^2^ values. The *I*^2^ refers to the percentage of variation across studies that is due to heterogeneity rather than chance, and does not inherently depend on the number of studies considered [*I*^2^ = 100% × (*Q* − df)/*Q*]. We evaluated the cutoff values for *I*^2^s for assignment of low (<25%), moderate (25–50%), and high (>50%) heterogeneities. If the *I*^2^ value was >50%, subgroup analysis was done.^[74]^ Sensitivity analyses were performed to examine the influence of each study on the pooled prevalence rate, OR, or HR, by serially omitting an individual study and pooling the remaining studies. Publication bias was examined by funnel plots, and Egger tests were employed for evaluating the degree of asymmetry. A *P*-value of <.1 was designated as an indicator of publication bias. Pooled analysis was performed using Comprehensive meta-analysis software (version 2.0; Biostat, Englewood, NJ).

## 3 Results

Sixty-nine studies with a total of 5736 cases were included in this meta-analysis (Fig. 1).^[3–71]^ The main features of the chosen studies are described in Table 1. The number of patients in each study was between 4 and 541.

**Table 1:**
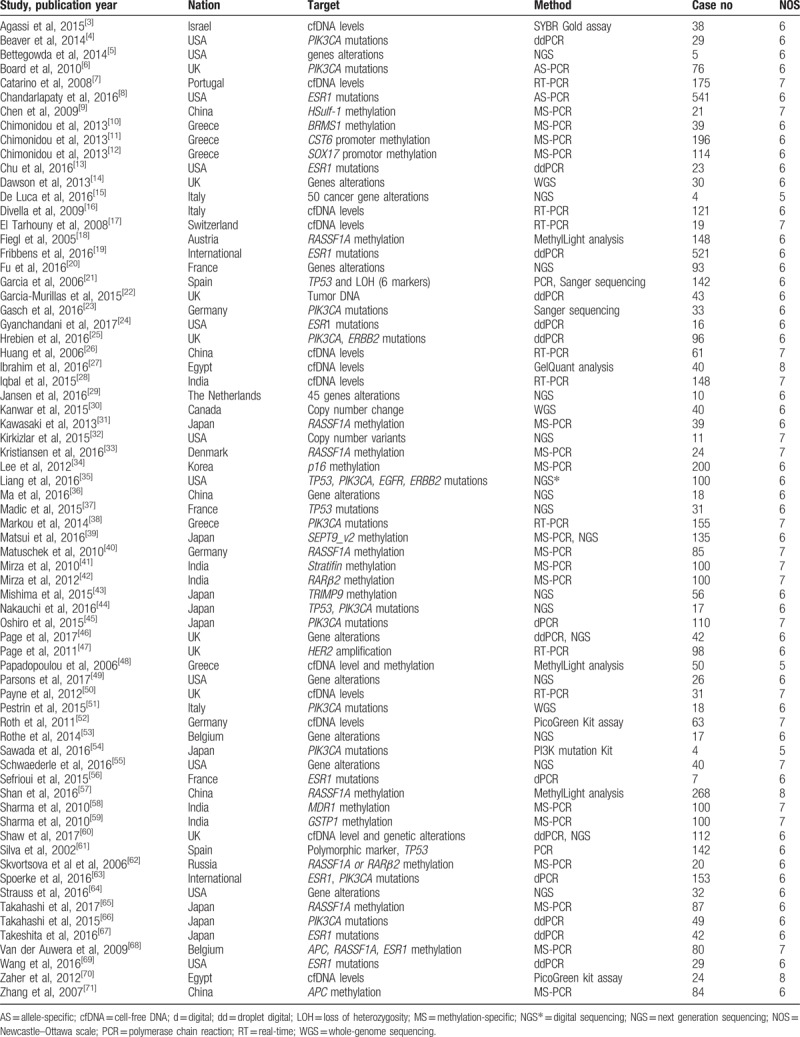
Characteristics of liquid biopsy studies with circulating tumor DNA or cell-free DNA in breast cancer.

### 3.1 Cancer screening and early detection

The pooled analysis of 37 studies on 2748 patients with BC revealed that the overall prevalence rate of ctDNA mutations in patients with BC was 44.3% (95% CI: 38.2–50.6%) (Supplementary Table 1). The ctDNA mutation frequencies of the *TP53*, *PIK3CA*, and *ESR1* genes were 37.8% (95% CI: 25.4–52.1%), 26.6% (22.4–31.2%) and 32.4% (26.8–38.5%) in the 11,19, and 10 studies consisting of 338, 1015 and 1379 patients with BC, respectively.^[4,6,8,13,19,23–25,29,35–38,44–46,49,53–56,60,63,64,66,67,69]^ Among the studies that presented the frequency of ctDNA mutations in the *TP53* and *PIK3CA* genes, eleven and nineteen studies were able to distinguish between patients with early surgery and patients with metastasis. However, The *TP53* and *PIK3CA* mutation rates were not different between the early operable patients and the advanced patients with BC (Q = 0.553, *P* = .57; and Q = 0.160, *P* = .689, respectively). Surprisingly, the detection rate of ctDNA mutations using next generation sequencing (NGS) techniques was 100% in patients with BC regardless of target genes.^[5,15,36]^

The pooled analysis of 21 studies involving 2046 patients with BC showed that the overall prevalence rate of ctDNA hypermethylation in patients with BC was 32.8% (95% CI: 26.8–39.4%) (Supplementary Table 2). However, the prevalence of ctDNA hypermethylation was significantly lower than that of ctDNA mutations in patients with BC (32.8% vs. 44.3%, *P* = .013).

**Figure 2:**
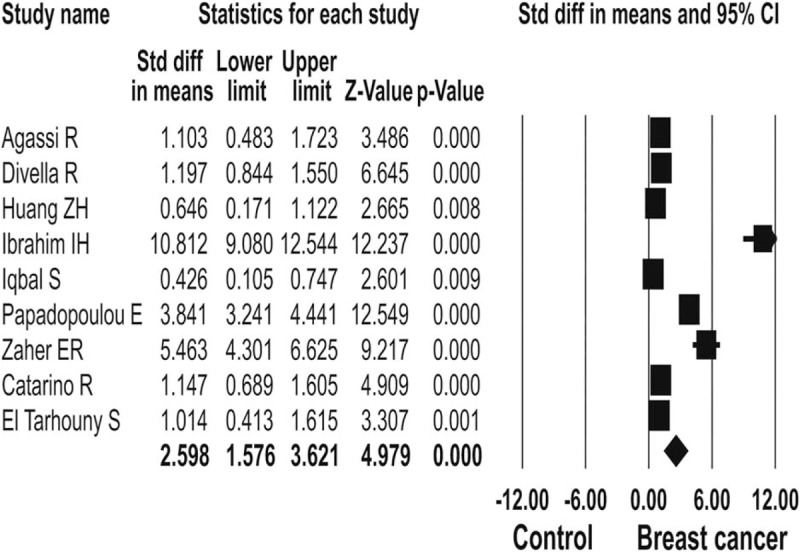
Weighed mean differences with corresponding 95% confidence intervals (CIs) of individual studies and pooled data, in cell-free DNA levels, between healthy controls and patients with breast cancer. The forest plot depicts each study and overall effect sizes and 95% CIs.

Nine studies compared cfDNA levels between healthy controls and patients with BC (Supplementary Table 3). The cfDNA levels were significantly higher in patients with BC than in healthy controls (WMD = 2.598; 95% CI: 1.576–3.621; *P* < .001, *Q* = 271.821, *I*^2^ = 97.057) (Fig. 2). To explore potential heterogeneity sources, we performed subgroup analyses according to the detection methods and ethnicity. The cfDNA levels were measured by real-time PCR, GelQuant, PicoGreen, and SYBR Gold assay. The WMD of each group according to the test methods was 1.36, 10.81, 5.46, and 1.10, respectively. Since the *P*-value of *Q* test for the group differences was <.001, we could reject the null hypothesis that the effect sizes between groups are the same. The subgroup group variable (detection methods) could account for 57.7% of the total actual variance (*R*^2^ = 0.577). In addition, the patients were classified into African, Asian, and Caucasian. The WMD of each group according to the ethnicity was 7.78, 0.54, and 1.65, respectively. Similar to the detection method variable, we could reject the null hypothesis that the effect sizes between groups are the same because the *P*-value of *Q* test for the group differences was <.001. The ethnicity of moderator variable could account for 55.3% of the total actual variance (*R*^2^ = 0.577) (Supplementary Table 4). Thus, different cfDNA detection methods and ethnic differences were considered potential sources of heterogeneity.

### 3.2 Axillary lymph node metastasis and clinical staging

Four studies described the relationship between ctDNA mutations and axillary lymph node metastasis in 217 patients with BC.^[4,23,45,66]^ No relationship was found between ctDNA mutations and axillary lymph node metastasis (OR = 1.764; 95% CI: 0.877–3.548; *P* = .112, *Q* = 3.283, *I*^2^ = 8.609). Eight studies investigated the relationship between ctDNA hypermethylation and axillary lymph node metastasis in 819 patients with BC.^[10,12,18,41,42,58,59,71]^ There was no association between ctDNA hypermethylation and axillary lymph node metastasis (OR = 0.829; 95% CI: 0.563–1.220; *P* = .341, *Q* = 9.810, *I*^2^ = 28.646).

**Figure 3:**
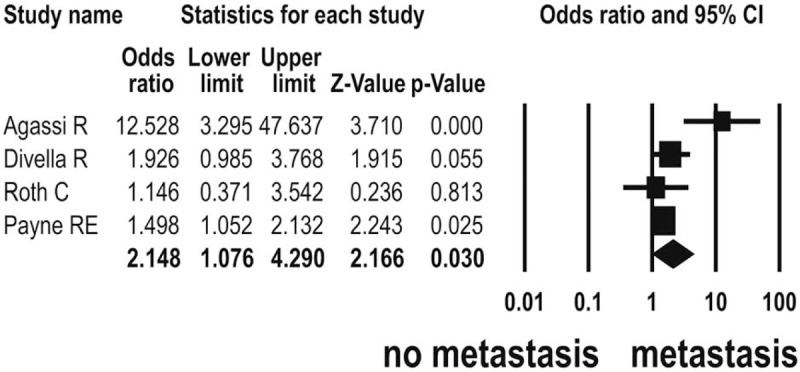
Odds ratios with corresponding 95% confidence intervals (CIs) of individual studies and pooled data for the association between cell-free DNA levels and axillary lymph node metastasis in breast cancer. The forest plot depicts each study and overall effect sizes and 95% CIs.

Four studies addressed the association between cfDNA levels and axillary lymph node metastasis in 277 patients with BC (Supplementary Tables 5 and 6). The cfDNA level was significantly associated with axillary lymph node metastasis (OR = 2.148; 95% CI: 1.076–4.290; *P* = .030, *Q* = 9.685, *I*^2^ = 69.023) (Fig. 3). To explore potential sources of heterogeneity, we conducted a subgroup analysis based on the detection methods. The cfDNA levels were measured by real-time PCR, SYBR Gold, and PicoGreen assays. The OR of each group according to the test methods was 1.58, 12.54, and 1.14, respectively. Since the *P*-value for *Q* test for the group differences was 0.01, we could reject the null hypothesis that the effect sizes between groups are the same. The subgroup group variable (detection methods) could account for 100% of the total actual variance (*R*^2^ = 1.0) (Supplementary Table 6).

Two studies reported the association between ctDNA mutations and clinical stage (I–II vs III–IV) in 40 patients with BC.^[23,24]^ There was no association between ctDNA mutations and clinical stage (OR = 0.942; 95% CI: 0.187–4.753; *P* = .943, *Q* = 1.251, *I*^2^ = 20.050). Five studies addressed the relationship between ctDNA hypermethylation and clinical stage in 487 patients with BC.^[41,42,58,59,65]^ No association was found between ctDNA hypermethylation and clinical stage (OR = 1.095; 95% CI: 0.444–2.703; *P* = .844, *Q* = 19.840, *I*^2^ = 79.839).

### 3.3 Cancer recurrence and patient survival

**Figure 4:**
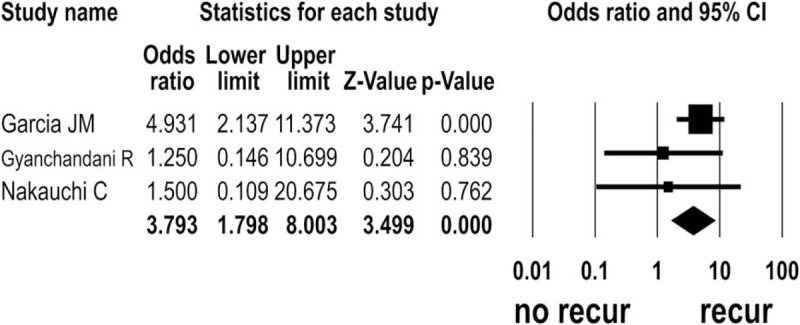
Odds ratios with corresponding 95% confidence intervals (CIs) of individual studies and pooled data for the association between circulating tumor DNA mutations and tumor recurrence in breast cancer. The forest plot depicts each study and overall effect sizes and 95% CIs.

Cancer recurrence was defined as locoregional relapse and distant metastasis after initial treatments. This included cases with recurrence locally in the ipsilateral breast or chest wall, regionally in drainage lymph nodes, or at distant sites.^[21,44]^ Three studies evaluated the association between ctDNA mutations and cancer recurrence in patients with BC.^[21,24,44]^ Genetic aberrations in ctDNA were more frequent in 34 (59%) of patients with 58 BC with recurrence than in 39 (33%) of 117 patients with BC without recurrence. The detection rate of ctDNA mutations was significantly associated with tumor recurrence (OR = 3.793; 95% CI: 1.798–8.003; *P* < .001, *Q* = 1.885, *I*^2^ = 0.000) (Fig. 4).

**Figure 5:**
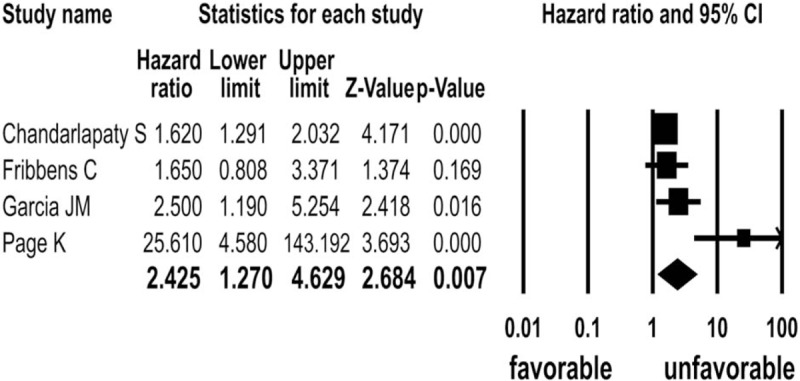
Hazard ratios with corresponding 95% confidence intervals (CIs) of individual studies and pooled data for the association between circulating tumor DNA mutations and overall survival outcomes in breast cancer. The forest plot depicts each study and overall effect sizes and 95% CIs.

Four studies presented hazard ratios (HRs) and CIs of overall survival outcomes in 782 patients with BC according to genetic mutations in ctDNA.^[8,19,21,46]^ The unadjusted HRs ranged from 1.62 to 25.61. The ctDNA mutations were significantly associated with poor overall survival outcomes in patients with BC (HR = 2.425; 95% CI: 1.270–4.629; *P* = .007, *Q* = 10.693, *I*^2^ = 71.945) (Fig. 5). We conducted the subgroup analyses based on the mutated gene types (*ESR1* mutations vs the other ctDNA mutations; *Q* = 0.006, *P* = .941) and ethnicity (Caucasian vs the other races; *Q* = 0.469, *P* = .493). However, since the *P*-values for *Q* tests for the group differences were >.1, we could not reject the null hypothesis that the effect sizes between groups are the same (Supplementary Table 7).

Two studies presented HRs and CIs of univariate disease-free survivals in 185 patients with BC according to ctDNA mutations.^[21,22]^ The detection of ctDNA mutations in patients with BC was significantly associated with short univariate disease-free survival outcomes (HR = 5.180; 95% CI: 1.215–22.084; *P* = .026, *Q* = 3.934, *I*^2^ = 74.578). Three studies described HRs and CIs of multivariate disease-free survivals in 325 patients with BC according to ctDNA mutations.^[21,22,26]^ The adjusted HRs ranged from 2.6 to 9.6. The ctDNA mutation rate in patients with BC was significantly associated with short multivariate disease-free survival rates (HR = 3.605; 95% CI: 1.718–7.562; *P* = .001, *Q* = 2.567, *I*^2^ = 22.078).

Three studies described HRs and CIs of progression-free survivals in 597 patients with BC according to ctDNA mutations.^[8,19,63]^ The detection of ctDNA mutations in patients with BC was significantly associated with shorter progression-free survival (HR = 1.311; 95% CI: 1.060–1.621; *P* = .013, *Q* = 1.089, *I*^2^ = 0.000). However, the survival data of investigations using ctDNA hypermethylation and cfDNA levels were insufficient to perform a meta-analysis.

### 3.4 Sensitivity analyses and publication bias

**Figure 6:**
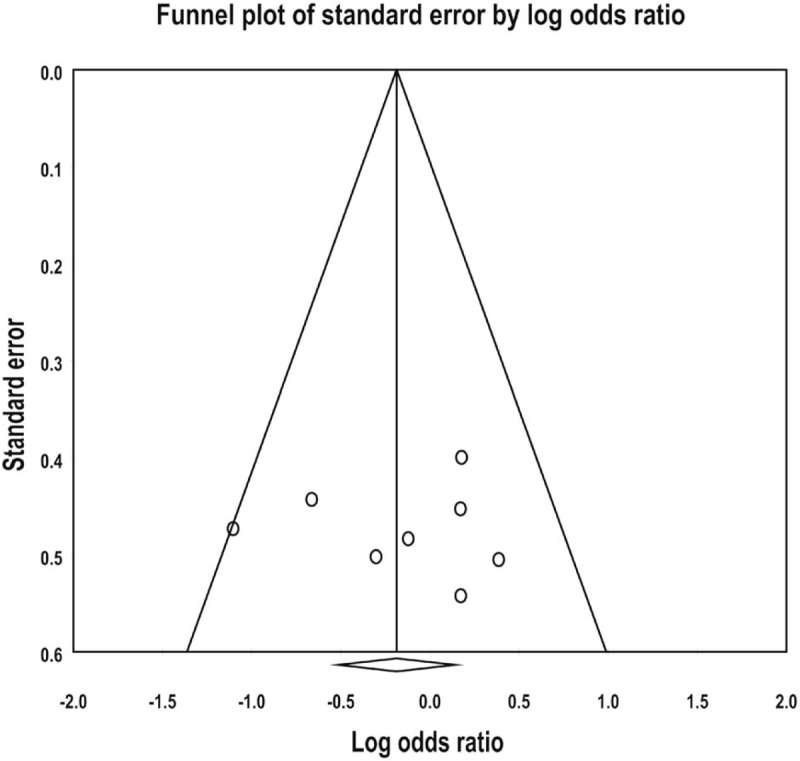
Funnel plot of meta-analysis for the association between circulating tumor DNA hypermethylation and axillary lymph node metastasis in breast cancer. Individual studies, indicated by small circles, are uniformly distributed in an inverted V-shape, and indicate no publication bias.

The sensitivity analyses showed that the study by Roth et al^[52]^ affected the pooled OR between cfDNA levels and axillary lymph node metastasis. If the Roth's study was removed, the summary effect size would be increased from 2.148 to 3.611. None of the other sensitivity analyses affected the summary effect sizes. The funnel plots and Egger regression tests revealed no evidence of publication bias (Fig. 6), except for the meta-analysis of the association between cfDNA levels and the prevalence of patients with BC (Supplementary Table 8).

## 4 Discussion

This meta-analysis was conducted to estimate the clinical usefulness of liquid biopsies to predict axillary lymph node metastasis, cancer recurrence, and patient's survival in BC. The pooled analysis of 69 studies with a total of 5736 cases revealed that ctDNA mutations are useful prognostic markers for recurrence and survival in patients with BC. In addition, cfDNA levels can be predictive of axillary lymph node metastasis.

For the early detection of BC, cfDNA levels and ctDNA mutations of *TP53*, *PIK3CA*, and *ESR1* genes were not valuable as biomarkers, although the ctDNA mutations were frequently detected in patients with BC. There was no significant difference in mutation frequencies of *TP53* and *PIK3CA* ctDNA between early and advanced patients with BC. The mutations of *TP53* and *PIK3CA* are often selected for liquid biopsy in patients with BC, because they are the 2 most common genetic variants in newly diagnosed BCs.^[44]^ Since the 2 genes are mutually exclusive, the prevalence of mutations in at least one of these genes in all patients with BC increases from 55% to 65%.^[44]^ Moreover, these mutations are generally concentrated in the exons 5 to 9 of TP53 and exon 9/20 of PIK3CA and are conserved between primary tumors and recurrent or metastatic cancers.^[44]^ Advances in NGS have dramatically improved the detection rates of ctDNA mutations in blood.^[5,13,15,32,49]^ The development of a BC-specific gene panel of ctDNA mutations using NGS technology is expected to enable clinicians to detect BC early. In contrast, although cfDNA levels were higher in patients with BC than in healthy controls, there was considerable overlap between the groups. Furthermore, there was a significant variation in cfDNA levels depending on the detection methods. As a marker for early detection of BC, the possibility of cfDNA level testing is reduced.

More recently, the *ESR1* mutations have been associated with as a resistance mechanism for endocrine therapy, which are clustered in the ligand-binding domain of the receptor, and result in ligand-independent estrogen receptor activity.^[24,75]^ The *ESR1* mutations are rare in primary patients with BC without metastasis, but are more commonly found in patients with metastatic BC or metastatic BC after endocrine therapy.^[75]^ In this meta-analysis, the *ESR1* mutations were analyzed only in patients with advanced or metastatic BC. Given that *ESR1* ctDNA mutations are generally associated with endocrine therapy resistance and poor survival, the mutated *ESR1* can be explored as a therapeutic target.

The results of our analysis showed that high cfDNA levels were significantly associated with axillary lymph node metastasis. Conservative approach of axillary staging has been developed in patients with BC such as the examination of a sentinel node, which is the first lymph node draining from a tumor bed. Sentinel node biopsy during surgery is important for staging the status of axillary lymph node involvement. Therefore, the prediction of preoperative axillary lymph node status as well as sentinel node biopsy during surgery is important determinants for postoperative treatments of BC. There has been considerable controversy as to whether ctDNA mutations or high levels of cfDNA could predict axillary lymph node metastasis. In some studies, high levels of cfDNA or ctDNA hypermethylation have been shown to be associated with lymph node metastasis or advanced clinical stage.^[3,50,52,65]^ In contrast, Mirza et al^[41]^ and Sharma et al^[58]^ reported that ctDNA hypermethylation was significantly associated with a low probability of lymph node metastasis and low clinical stages. However, this meta-analysis showed that ctDNA mutations and hypermethylation are not associated with lymph node metastasis or clinical stage. Our meta-analysis has demonstrated a significant prognostic value of ctDNA mutations in tumor recurrence and poor survival in patients with BC. The presence of ctDNA mutations was significantly related to tumor recurrence with a 4-fold OR. Of 58 patients with BC recurrence, 19 (33%) had local recurrence and 39 (67%) had metastatic recurrence.

Unfortunately, because there was not enough data on this aspect, a meta-analysis could not be performed to assess whether ctDNA mutations were associated with local recurrence or remote recurrence. The ctDNA mutation rate was a prognostic indicator of reduced disease-free survival and overall survival with 2-fold and 4-fold HRs, respectively. Thus, this meta-analysis suggests that liquid biopsy may be useful as a decision-making tool for postoperative adjuvant chemotherapy in patients with localized BC.

There are several limitations in this meta-analysis. First, individual primary studies used different target genes, various analytical methods, and heterogeneous clinical samples. Since ctDNA and cfDNA detection methods are very different, we performed pool analysis for each. Second, of the studies included in the meta-analysis, ctDNA and cfDNA tests were recently recognized and developed only as potential biomarkers, so small sample size studies were included. Many of these small studies can distort the results of meta-analysis. In addition, in patients with cancer, ctDNA is a fraction of cfDNA, accounting for approximately 0.01% to as much as 50%, so that there is a limitation that they cannot be completely separable from each other.^[76]^ However, most cfDNAs in healthy individuals are originate from bone marrow with a length of 70 to 200 bp and a concentration of 0 to 100 ng/mL. In contrast, ctDNAs of patients with cancer are 200 bp to >1 kb in length and the half-life is 15 minutes to several hours, which are removed from the liver and kidneys.^[76]^ There is a need to develop laboratory methods to make it easier to distinguish clinically from cfDNA and ctDNA. Finally, we have classified the patient as white, Asia or Africa for subgroup analysis, but in reality there is a possibility of discrepancies between our classification and the original data. These limitations might affect the results of this pooled analysis.

In conclusion, this meta-analysis provides evidence that ctDNA mutations are significantly associated with tumor recurrence and poor survival outcomes in patients with BC. In addition, high cfDNA levels may be indicators of axillary lymph node metastasis, which is an important determinant for postoperative adjuvant chemotherapy.

## Author contributions

**Conceptualization:** Young-Sik Kim.

**Data curation:** Ju-Han Lee, Young-Sik Kim.

**Formal analysis:** Ju-Han Lee, Hoiseon Jeong, Jung-Woo Choi, HwaEun Oh.

**Funding acquisition:** Young-Sik Kim.

**Investigation:** Ju-Han Lee.

**Methodology:** Hoiseon Jeong, Jung-Woo Choi, HwaEun Oh.

**Supervision:** Young-Sik Kim.

**Validation:** Hoiseon Jeong, Jung-Woo Choi, HwaEun Oh, Young-Sik Kim.

**Writing – original draft:** Ju-Han Lee.

**Writing – review & editing:** Young-Sik Kim.

## Supplementary Material

Supplemental Digital Content
