# Supplementary material for: Liquid biopsy prediction of axillary lymph node metastasis, cancer recurrence, and patient survival in breast cancer: A meta-analysis
Source: Medicine (Baltimore). 2018 Oct 19;97(42):e12862. doi: 10.1097/MD.0000000000012862 (PMC6211877; doi:10.1097/MD.0000000000012862)
Supplement: Supplemental Digital Content [file medi-97-e12862-s001.doc]

Supplementary Table S1.Individual studies on circulating tumor DNA mutations

| Study | Mutated/Total (%) | *TP53*/Total (%) | *PIK3CA*/Total (%) | *ESR1*/Total (%) |
| --- | --- | --- | --- | --- |
| Beaver JA ^[4]^ | 13/29 (44.8) | NA | 13/29 (44.8) | NA |
| Bettegowda C ^[5]^ | 5/5 (100.0) | NA | NA | NA |
| Board RE^6^ | 13/76 (17.1) | NA | 13/76 (17.1) | NA |
| Chandarlapaty S ^[8]^ | 156/541 (28.8) | NA | NA | 156/541 (28.8) |
| Chu D ^[13]^ | 15/23 (65.2) | NA | NA | 15/23 (65.2) |
| Dawson SJ ^[14]^ | 29/30 (96.7) | NA | NA | NA |
| De Luca F ^[15]^ | 4/4 (100.0) | NA | NA | NA |
| Fribbens C ^[19]^ | 154/521 (29.6) | NA | NA | 154/521 (29.6) |
| Fu Y ^[20]^ | 50/93 (53.8) | NA | NA | NA |
| Garcia JM ^[21]^ | 61/142 (43.0) | NA | NA | NA |
| Garcia-Murillas ^[22]^ | 13/43 (13.2) | NA | NA | NA |
| Gasch ^[23]^ | 12/33 (36.4) | NA | 12/33 (36.4) | NA |
| Gyanchandani ^[24]^ | 5/16 (31.3) | NA | NA | 5/16 (31.3) |
| Hrebien ^[25]^ | 42/96 (43.8) | NA | 22/96 (22.9) | NA |
| Jansen MP ^[29]^ | 6/10 (60.0) | 2/10 (20.0) | 1/10 (10.0) | NA |
| Kanwar N ^[30]^ | 17/40 (42.5) | NA | NA | NA |
| Kirkizlar E ^[32]^ | 8/11 (72.7) | NA | NA | NA |
| Liang DH ^[35]^ |  | 27/100 (27.0) | 22/100 (22.0) | NA |
| Ma F ^[36]^ | 18/18 (100.0) | 8/18 (44.4) | 7/18 (38.9) | NA |
| Madic J ^[37]^ | 22/31 (71.0) | 22/31 (71.0) | NA | NA |
| Markou A ^[38]^ | 34/155 (21.9) | NA | 34/155 (21.9) | NA |
| Nakauchi C ^[44]^ | 7/17 (41.2) | 5/17 (29.4) | 5/17 (29.4) | NA |
| [Oshiro C](https://www.ncbi.nlm.nih.gov/pubmed/?term=Oshiro C%5BAuthor%5D&cauthor=true&cauthor_uid=25736040) ^[45]^ | 25/110 (22.7) | NA | 25/110 (22.7) | NA |
| Page K ^[46]^ | 21/42 (50.0) | 6/42 (14.3) | 12/42 (28.6) | 6/42 (14.3) |
| Page K ^[47]^ | 13/98 (13.3) | NA | NA | NA |
| Parsons HA ^[49]^ | 24/26 (92.3) | 21/26 (80.8) | 3/26 (11.5) | NA |
| Pestrin M ^[51]^ | 6/18 (33.3) | NA | NA | NA |
| Rothé F ^[53]^ | 12/17 (70.6) | 5/17 (29.3) | 5/17 (29.3) | NA |
| Sawada T ^[54]^ | 1/4 (25.0) | NA | 1/4 (25.0) | NA |
| Schwaederle M ^[55]^ | 28/40 (70.0) | 13/40 (32.5) | 10/40 (25.0) | NA |
| Sefrioui D ^[56]^ | 4/7 (57.1) | NA | NA | 4/7 (57.1) |
| Shaw JA ^[60]^ | 4/5 (80.0) | 2/5 (40.0) | 3/5 (60.0) | 3/5 (60.0) |
| Silva JM ^[61]^ | 61/142 (43.0) | NA | NA | NA |
| Spoerke JM ^[63]^ | 85/153 (55.6) | NA | 62/156 (39.7) | 57/153 (37.3) |
| Strauss WM ^[64]^ | 18/32 (56.3) | 11/32 (34.4) | 6/32 (18.8) | NA |
| Takeshita T ^[66]^ | 12/49 (24.5) | NA | 12/49 (24.5) | NA |
| Takeshita T ^[67]^ | 12/42 (28.6) | NA | NA | 12/42 (28.6) |
| Wang P ^[69]^ | 7/29 (24.1) | NA | NA | 7/29 (24.1) |
| Overall  (n = 37) | 1017/2748  (44.3*) | 122/338  (37.8*) | 268/1015 (26.6*) | 419/1379  (32.4*) |

*: overall prevalence of each estimated by meta-analysis using a random-effects model; NA: not available.

Supplementary Table S2. Individual studies on circulating tumor DNA hypermethylation

| Study | Hypermethylated gene | Hypermethylated case/Total (%.) |
| --- | --- | --- |
| Chen Z ^[9]^ | *HSulf-1* | 16/21 (76.2) |
| Chimonidou M ^[10]^ | *BRMS1* | 5/39 (12.8) |
| Chimonidou M ^[11]^ | *CST6* promoter | 63/196 (32.1) |
| Chimonidou M ^[12]^ | *SOX17*promoter | 43/114 (37.7) |
| Fiegl H ^[18]^ | *RASSF1A* | 33/148 (22.3) |
| Kawasaki H ^[31]^ | *RASSF1A* | 11/39 (28.2) |
| Kristiansen S ^[33]^ | *RASSF1A* | 20/24 (83.3) |
| Lee JJ ^[34]^ | *p16* | 69/200 (34.5) |
| Matsui S ^[39]^ | *SEPT9_v2* | 35/135 (25.9) |
| Matuschek C ^[40]^ | *RASSF1A* | 22/85 (25.9) |
| Mirza S ^[41]^ | *Stratifin* | 56/100 (56.0) |
| Mirza S ^[42]^ | *RARβ2* | 20/100 (20.0) |
| Mishima C ^[43]^ | *TRIMP9* | 10/56 (17.9) |
| Papadopoulou E ^[48]^ | *RASSF1A* | 13/50 (26.0) |
| Shan M ^[57]^ | *RASSF1A* | 46/268 (17.2) |
| Sharma G ^[58]^ | *MDR1* | 56/100 (56.0) |
| Sharma G ^[59]^ | *GSTP1* | 26/100 (26.0) |
| Skvortsova TE ^[62]^ | *RASSF1A or RARβ2* | 6/20 (30.0) |
| Takahashi H ^[65]^ | *RASSF1A* | 20/87 (23.0) |
| Van der Auwera I ^[68]^ | *APC or RASSF1A or ESR1* | 42/80 (52.5) |
| Zhang JJ ^[71]^ | *APC* | 26/84 (31.0) |
| Overall (n = 21) |  | 638/2046 (32.8*) |

*: overall prevalence estimated by meta-analysis using a random-effects model.

Supplementary Table S3. Individual studies on cell-free DNA levels between healthy controls and breast cancer patients

|  |  |  | Healthy control | | |  | Cancer | | |  |  |
| --- | --- | --- | --- | --- | --- | --- | --- | --- | --- | --- | --- |
|  | Targeted DNA | Method | Case no. | Mean | SD |  | Case no. | Mean | SD | P | OR (95% CI) |
| Agassi R ^[3]^ | DNA (260nm) | SYRB Gold assay | 16 | 395 | 248 |  | 38 | 1010 | 642 | <0.001 | NA |
| Divella R ^[16]^ | *hTERT* | RT- PCR | 50 | 0.03 | 0.09 |  | 121 | 0.98 | 0.94 | <0.001 | NA |
| Huang ZH ^[26]^ | *β-globin* | RT- PCR | 25 | 12.9 | 10.6 |  | 61 | 64.6 | 94.4 | NA | NA |
| Ibrahim IH ^[27]^ | DNA (260nm) | GelQuant analysis | 40 | 19.6 | 5.4 |  | 40 | 335 | 40.9 | <0.05 | NA |
| Iqbal S ^[28]^ | *ALU247* | RT-PCR | 51 | 11.4 | 9.01 |  | 148 | 57.4 | 124.88 | NA | NA |
| Papadopoulou E ^[48]^ | *β-globin* | RT- PCR | 54 | 11.4 | 9.5 |  | 68 | 45.8 | 8.5 | NA | NA |
| Zaher ER ^[70]^ | DNA (260nm) | PicoGreen Kit assay | 30 | 75.8 | 20.8 |  | 24 | 902 | 226.2 | NA | NA |
| [El Tarhouny S ^[17^](https://www.ncbi.nlm.nih.gov/pubmed/?term=El Tarhouny S%5BAuthor%5D&cauthor=true&cauthor_uid=18691902)] | *GAPDH* | RT- PCR | 32 | 12,796 | NA |  | 19 | 38,180 | NA | 0.001 | NA |
| Catarino R ^[7]^ | *hTERT* | RT- PCR | 80 | NA | NA |  | 175 | NA | NA | NA | 8.01 (3.49-18.38) |

SD: standard deviation; OR: odds ratio; CI: confidence interval; NA: not available; RT-PCR: real-time polymerase chain reaction.

Supplementary Table S4. Subgroup analysis of cell-free DNA levels between healthy controls and breast cancer patients

|  | Effect size | | |  | T^2^ |  |  |
| --- | --- | --- | --- | --- | --- | --- | --- |
| Category | Study No. | WMD (95% CI) | P | P_Q_ | Between | Overall | R^2^ |
| Overall WMD before subgroup analysis | 9 | 2.598 (1.576 - 3.621) | <0.001 |  |  |  |  |
| Detection method |  |  |  | < 0.001 | 0.969 | 2.293 | 0.577 |
| Real-time PCR | 6 | 1.361 (0.550-2.172) | 0.001 |  |  |  |  |
| GelQuant assay | 1 | 10.812 (8.220-13.404) | <0.001 |  |  |  |  |
| PicoGreen Kit | 1 | 5.463 (3.211-7.715) | <0.001 |  |  |  |  |
| SYRB Gold assay | 1 | 1.103 (-0.923-3.129) | 0.286 |  |  |  |  |
|  |  |  |  |  |  |  |  |
| Ethnicity |  |  |  | < 0.001 | 1.024 | 2.293 | 0.553 |
| African | 2 | 7.776 (6.045 - 9.508) | <0.001 |  |  |  |  |
| Asian | 2 | 0.535 (-0.897 - 1.966) | 0.464 |  |  |  |  |
| Caucasian | 5 | 1.652 (0.734 - 2.571) | <0.001 |  |  |  |  |

T^2^: Tau-squared; WMD: weighted mean difference; CI: confidence interval; P: P value for weighted mean difference; P_Q_: P value for Q test; R^2^: R-squared, the ratio of explained variance to total variance.

Supplementary Table S5. Individual studies on the association between cell-free DNA levels and axillary lymph node metastasis

|  |  |  |  | No LN metastasis | | |  | LN metastasis | | |  |
| --- | --- | --- | --- | --- | --- | --- | --- | --- | --- | --- | --- |
|  | Targeted DNA | Method | Odds ratio (95% CI) | Case | Mean | SD |  | Case | Mean | SD | P |
| Agassi R ^[3]^ | DNA (260nm) | SYRB Gold | 12.528 (3.295-47.637) | 23 | 734 | 451 |  | 14 | 1486 | 663 | <0.01 |
| Divella R ^[16]^ | *hTERT*gene | Real-time PCR | 1.926 (0.985-3.768) | 46 | 0.8 | 0.56 |  | 75 | 1.08 | 0.88 | - |
| Payne RE ^[50]^ | GAPDH gene | Real-time PCR | 1.498 (1.052-2.132) | 22* | 2.66 | - |  | 42* | 4.35 | - | 0.025 |
| Roth C ^[52]^ | DNA (260nm) | PicoGreen Kit | 1.146 (0.371-3.542) | 13 | 8027 | 518 |  | 42 | 8079 | 737 | - |

LN: lymph node; OR: odds ratio; CI: confidence interval; SD: standard deviation; P: P value; *:the study used 149 and 322 samples from non-metastatic and metastatic patients, respectively.

Supplementary Table S6. Subgroup analysis of cell-free DNA levels between axillary lymph node non-metastatic and metastatic groups

|  | Effect size | | |  | T^2^ | |  |
| --- | --- | --- | --- | --- | --- | --- | --- |
| Category | Study no. | OR (95% CI) | P | P_Q_ | Between | Overall | R^2^ |
| Overall OR before subgroup analysis | 4 | 2.148 (1.076 – 4.290) | 0.030 |  |  |  |  |
| Detection method |  |  |  | 0.010 | 0.000 | 0.315 | 1.000 |
| Real-time PCR | 2 | 1.582 (1.157 – 2.162) | 0.004 |  |  |  |  |
| SYRB Gold assay | 1 | 12.528 (3.295 – 47.637) | <0.001 |  |  |  |  |
| PicoGreen Kit | 1 | 1.146 (0.371 – 3.542) | 0.813 |  |  |  |  |

T^2^: Tau-squared; no.; number; OR: odds ratio; CI: confidence interval; P: P value for odds ratio; P_Q_: P value of Q statistics; R^2^: R-squared, the ratio of explained variance to total variance.

Supplementary Table S7. Subgroup analysis of ctDNA mutations on overall survival

|  | Effect size | | |  | T^2^ | |
| --- | --- | --- | --- | --- | --- | --- |
|  | Study no. | HR (95% CI) | P | P_Q_ | Between | Overall |
| Overall HR before subgroup analysis | 4 | 2.425 (1.270 - 4.629) | 0.007 |  |  |  |
| Gene |  |  |  |  |  |  |
| *ESR1* | 3 | 2.677 (1.057 - 6.784) | 0.038 | 0.941 | 0.482 | 0.278 |
| Others (Tumor DNA) | 1 | 2.500 (0.531 - 11.778), | 0.247 |  |  |  |
|  |  |  |  |  |  |  |
| Ethnicity |  |  |  | 0.493 | 0.574 | 0.278 |
| Caucasian | 3 | 3.236 (1.190 - 8.801) | 0.021 |  |  |  |
| International | 1 | 1.650 (0.318 - 8.574) | 0.551 |  |  |  |

T^2^: Tau-squared; no.: number; HR: hazard ratio; CI: confidence interval; P: P value for hazard ratio; P_Q_: P value for Q test

Supplementary Table S8.Egger’s tests for funnel plot asymmetry

| Meta-analysis | Regression intercept | P value |
| --- | --- | --- |
| cfDNA levels between healthy control and breast cancer | 11.838 | 0.005 |
| ctDNA mutations between non-LN metastatic and metastatic groups | -0.142 | 0.953 |
| ctDNA hypermethylation between non-LN metastatic and metastatic groups | 0.136 | 0.979 |
| cfDNA levels between non-LN metastatic and metastatic groups | 2.543 | 0.575 |
| ctDNA hypermethylation between low and high clinical stages | 15.316 | 0.101 |
| ctDNA mutations on recurrence | -1.623 | 0.159 |
| ctDNA mutations on overall survival | 2.2 | 0.187 |
| ctDNA mutations on disease-free survival* | 5.131 | 0.343 |
| ctDNA mutations on progression-free survival | -2.502 | 0.343 |

cfDNA: cell-free DNA; ctDNA: circulating tumor DNA; LN: lymph node; *; multivariate disease-free survival
